# Commentary: A Computational Theory of Mindfulness Based Cognitive Therapy from the “Bayesian Brain” Perspective

**DOI:** 10.3389/fpsyt.2021.575150

**Published:** 2021-02-02

**Authors:** Charles Verdonk, Marion Trousselard

**Affiliations:** ^1^Department Neurosciences and Cognitive Sciences, French Armed Forces Biomedical Research Institute, Brétigny-sur-Orge, France; ^2^French Military Health Service Academy, Paris, France; ^3^Lorraine University, APEMAC/EPSAM - EA 4360, Metz, France

**Keywords:** mindfulness, Bayesian brain, predictive coding, active inference, context-updating hypothesis

## Introduction

In their seminal paper, Manjaly and Iglesias (1) introduce a theoretical model of the neurocomputational underpinnings of Mindfulness Based Cognitive Therapy (MBCT). Taking a “Bayesian brain” perspective, they propose a promising framework that seeks to answer a question that remains at the frontier of neurosciences: “How does mindfulness work?”. The authors claim that mindful functioning increases the precision of likelihood (i.e., the precision of incoming sensory information), but decreases the precision of prior (i.e., the precision of internal model prior to receiving new sensory evidence), thus reducing the significance of prediction error (which is the discrepancy between the prior and the likelihood).

Manjaly and Iglesias propose that the being mode in mindfulness (i.e., accepting whatever sensations arise) may enhance the precision of likelihood by promoting attentional skills, notably the ability of focused attention, which enables individuals to access an extensive sensory experience (2). In our own recent work, we suggested that attentional amplification in mindfulness could result in a lower consciousness threshold, thus facilitating the access of sensory information to the global neural workspace [(3); for a review of the global neural workspace theory, see (4)]. Consequently, the increased quantity of (sensory or metacognitive) evidence that is consciously processed may improve the precision of information (likelihood).

**Figure 1:**
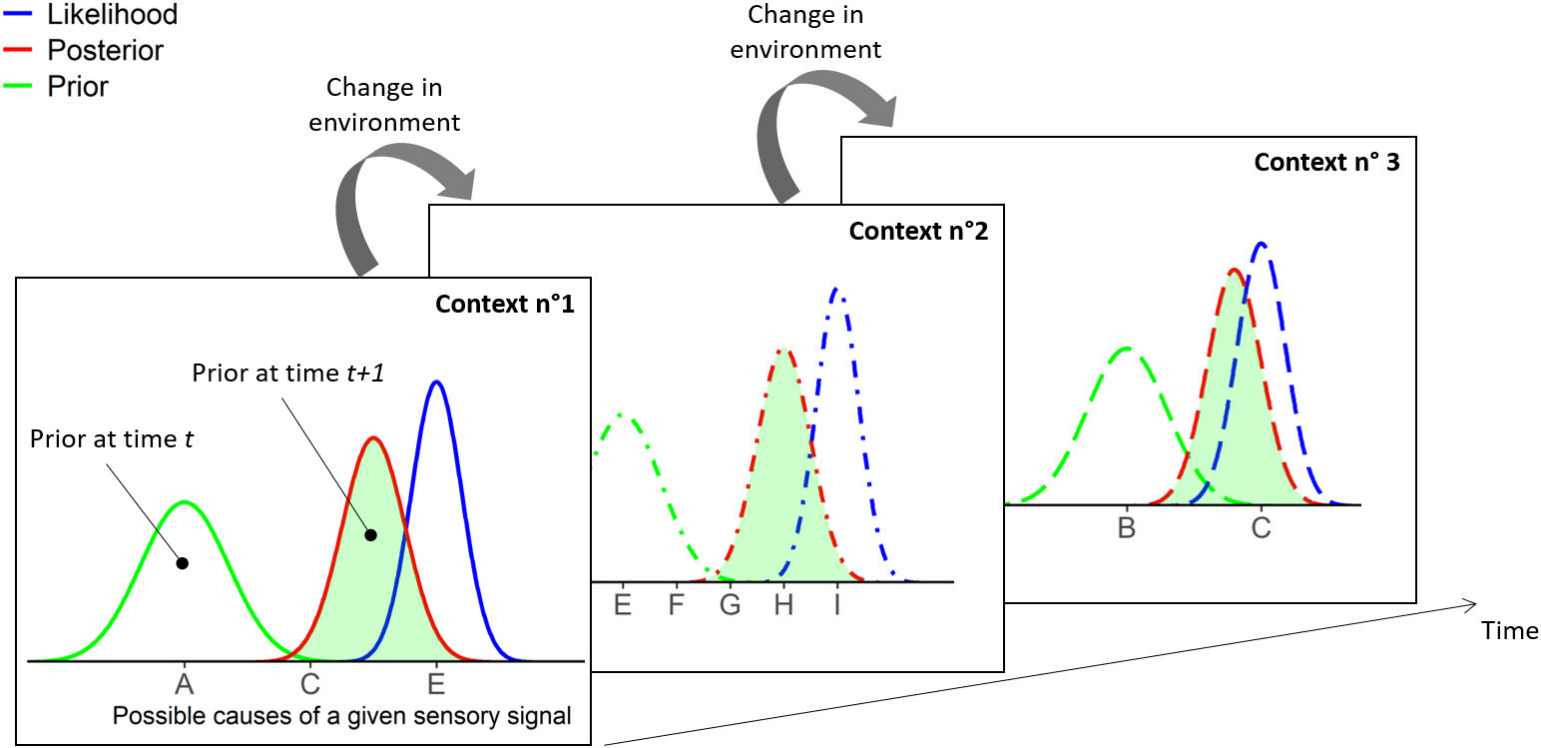
Graphical summary of two neurocomputational mechanisms through which mindfulness may work. First, mindfulness increases the precision of likelihood (i.e., the precision of incoming sensory information), by decreasing the threshold of conscious access through attentional amplification. Thus, at time *t*, belief is updated by integrating the more precise likelihood and the prior (i.e., the internal model prior to receiving new sensory evidence), which leads to a more precise posterior. Consequently, the newly updated prior at time *t*+*1* (green area), which is equivalent to the posterior at time *t*, shows an increased precision. Second, the attentional pattern of mindfulness, which is executed “moment to moment,” enables the prior belief to be optimally adjusted to the context of present experience. Thus, prior beliefs are iteratively updated as changes in environment occur over time. This dynamic process makes it possible to continuously minimize prediction error, which is the discrepancy between the prior and the likelihood, as the former is the best suited to the present context.

Nevertheless, we disagree on a second point, which argues that mindfulness could be associated with prior that is less precise (i.e., less informative), resulting in low reactivity (1). Even though such a computational mechanism could account for reduced reactivity (in the sense of active inference), we believe that it is clearly inconsistent with the first computational mechanism—the increased precision of likelihood. The brain is a dynamic system in which events are intrinsically dependent—one experience will impact the next. Thus, a more precise likelihood at time *t* is expected to lead to a more precise prior at time *t*+*1*. Indeed, the process of updating beliefs integrates new and old information (from present and past experience, respectively) to improve future predictions. Consequently, the precision of priors should gradually increase as mindful experience accumulates (Figure 1). It should be noted that, to keep behavior adaptive, the prior precision must not exceed the likelihood precision. Indeed, in cases in which prior becomes more precise than likelihood, one would expect that individuals become less adaptive because they are less inclined to change when receiving new information.

## The Context-Updating Hypothesis

Learning from (sensory) experience contributes to make an agent's prior (internal model) more precise, by reducing the range of possible causes of an input in relation to the context of experience. In other words, given an input and a context, possible causes are limited on the basis of contextual elements of past experience. Consequently, the probabilities of (remaining) causes increase, as well as the precision of prior, as experience accumulates.

Here, we argue that mindfulness is characterized by optimal adjustment of prior beliefs to the context of present experience, which contributes to minimizing prediction error. The attentional pattern of mindfulness, which is executed “moment to moment” (2, 5), could enable prior beliefs to be timely updated as a function of the present context.

Sensory information is continuously processed unconsciously. Conscious access, on the other hand, is thought to start when attention amplifies a given piece of information and allows it to access the global neural workspace (4). The context-updating theory suggests that a belief (mental representation) is updated when the individual receives a new piece of information indicating a change in the environment. After initial sensory processing, a process of comparison evaluates the representation of the previous context and, if new evidence is detected, the representation is updated (6). By linking the global neural workspace model and context-updating theory, we propose that the attentional pattern of mindfulness, which is executed “moment to moment,” may enable gradually more precise priors to be optimally updated as a function of the context of present, conscious experience. This dynamic, moment-to-moment process could help to minimize the prediction error by limiting the discrepancy between the likelihood and the prior, as the latter is expected to be the most appropriate given the present context. Our hypothesis may be represented graphically by iterative updating of prior beliefs as changes in environment occur over time (Figure 1). This dynamic process makes it possible to continuously minimize the distance between the likelihood and the prior, which reflects the significance of the prediction error.

This computational strategy is of particular interest in our ever-changing environment, because it enables continuous adjustment of cognitive and physiological reactivity. The flexibility that is cultivated through mindfulness practice could relate to this strategy when switching between modes (being vs. doing), depending on which has greater relevance to the present context. Returning to the clinical focus of Manjaly and Iglesias' article, depression relapse is thought to result from self-reinforcing rumination, which corresponds to repetitive, negative thoughts. Within the “context-updating” framework, rumination could be described as resulting from the lack of updating of the prior, which remains fixed on a negative mental representation (belief) irrespective of emotional changes in the present context (7). Furthermore, we suggest that the efficiency of MBCT in the treatment of depression relapse, including reduced rumination (8), could rely on better updating (flexibility) of priors as a function of the present, emotional context.

Manjaly and Iglesias provide several experimental recommendations for future empirical work testing their theoretical model. The context-updating hypothesis presented in this commentary can be tested using the hierarchical gaussian filter, which derives update equations for beliefs in considering their time-varying structure (9). Context flexibility (i.e., the ability to adjust belief as a function of current context) in mindfulness can be tested by estimating separate parameters (for priors, likelihood and prediction errors) on different temporal hierarchies. Finally, it should be noted that the context-updating framework may be applicable to other psychiatric disorders that can be seen in terms of a deficiency in context processing (i.e., context rigidity), such as the Post-Traumatic Stress Disorder (10) and schizophrenia (11).

## Conclusion

Manjaly and Iglesias propose a theoretical, computational framework that offers a promising way to investigate the mechanisms through which mindfulness improves health and well-being. However, it appears to us that a core mechanism in their model—namely, the decreased precision of prior—conflicts with the mechanism of increased precision of likelihood. We suggest that mindfulness could be characterized by the increased precision of prior, because of the increased precision of likelihood, and the dynamic updating of prior beliefs to the context of the present experience, which ultimately lead to optimal active inference.

## Author's Note

The opinions or assertions expressed herein are the private views of the authors and are not to be considered as official or as reflecting the views of the French Military Health Service.

## Author Contributions

Both authors listed have made a substantial, direct and intellectual contribution to the work, and approved it for publication.

## Conflict of Interest

The authors declare that the research was conducted in the absence of any commercial or financial relationships that could be construed as a potential conflict of interest.
